# An efficient and regioselective biocatalytic synthesis of aromatic *N*‐oxides by using a soluble di‐iron monooxygenase PmlABCDEF produced in the *Pseudomonas* species

**DOI:** 10.1111/1751-7915.13849

**Published:** 2021-06-11

**Authors:** Vytautas Petkevičius, Justas Vaitekūnas, Renata Gasparavičiūtė, Daiva Tauraitė, Rolandas Meškys

**Affiliations:** ^1^ Department of Molecular Microbiology and Biotechnology Institute of Biochemistry Life Sciences Center Vilnius University Saulėtekio 7 Vilnius LT‐10257 Lithuania

## Abstract

Here, we present an improved whole‐cell biocatalysis system for the synthesis of heteroaromatic *N*‐oxides based on the production of a soluble di‐iron monooxygenase PmlABCDEF in *Pseudomonas* sp. MIL9 and *Pseudomonas putida* KT2440. The presented biocatalysis system performs under environmentally benign conditions, features a straightforward and inexpensive procedure and possesses a high substrate conversion and product yield. The capacity of gram‐scale production was reached in the simple shake‐flask cultivation. The template substrates (pyridine, pyrazine, 2‐aminopyrimidine) have been converted into pyridine‐1‐oxide, pyrazine‐1‐oxide and 2‐aminopyrimidine‐1‐oxide in product titres of 18.0, 19.1 and 18.3 g l^‐1^, respectively. To our knowledge, this is the highest reported productivity of aromatic *N*‐oxides using biocatalysis methods. Moreover, comparing to the chemical method of aromatic *N*‐oxides synthesis based on *meta*‐chloroperoxybenzoic acid, the developed approach is applicable for a regioselective oxidation that is an additional advantageous option in the preparation of the anticipated *N*‐oxides.

## Graphical abstract

In this study, we describe a new whole‐cell biocatalysis system for aromatic *N*‐oxides synthesis based on production of soluble di‐iron monooxygenase PmlABCDEF. Various pyridine, pyrazine, and pyrimidine derivatives could be converted into appropriate *N*‐oxides on a preparative scale in the simple shake‐flask cultivation. This improved synthesis method was used to yield specific pyrazine and pyrimidine oxidation products that were difficult to obtain by employing a standard mCPBA‐based technique.

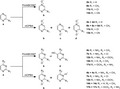

## Introduction

Aromatic *N*‐oxides (ArN→O) have been widely used as structural motifs in various fields due to their increased reactivity towards either electrophilic or nucleophilic agents compared to regular nitrogen heterocycles (Mfuh and Larionov, 2015). The appropriate *N*‐oxides of pyridines, pyrazines, pyrimidines and quinolines are precursors for a huge variety of C2‐functionalized bioactive compounds used in medicine today (Wang *et al*., 2016). No less important are *N*‐oxides themselves as *N* → O moiety is present in various pharmaceuticals, chemical catalysts, agriculture agents and pyrotechnic compounds, and they are promising units in future studies as well (Dyer *et al*., 2018). Quinoxaline 1,4‐di‐*N*‐oxide derivatives show a broad range of biological properties, including anti‐tubercular, antimicrobial, antitumoral, antitrypanosomal and antiinflammatory activities, thus possess potential applications in human and veterinary medicines (Srinivasarao *et al*., 2020). *In silico* molecular investigation showed that pyridine *N*‐oxide‐based antiviral compounds are potential inhibitors against human SARS infection, also they are more potent than common antiviral drugs chloroquine and hydroxychloroquine (Ghaleb *et al*., 2020). Chiral heteroaromatic *N*‐oxides have been intensively studied over the last few years as excellent organocatalysts for stereoselective reactions, including allylation, propargylation, allenylation, ring‐opening of *meso*‐epoxides and many more (Wrzeszcz and Siedlecka, 2020). Also, heterocyclic *N*‐oxide‐based compounds have been investigated as possible fluorogenic scaffolds that can be easily applied in the design and synthesis of small‐molecule fluorescent probes (Ma *et al*., 2020). However, the most common methods for ArN→O synthesis such as oxidation with *meta*‐chloroperoxybenzoic acid (*m*CPBA) (laboratory scale) or hydrogen peroxide (industrial scale) are hazardous and possess some serious limitations (Vörös *et al*., 2014). Also, ArN→O can be prepared by various rearrangements and ring‐closing reactions (Chucholowski and Uhlendorf, 1990; Nesi *et al*., 1992); nevertheless, the existing organic chemistry methods are not able to fully address existing demands and, usually, they do not match the increasing environmental requirements. Therefore, the establishment of biocatalytic methods for ArN→O production seems a promising alternative that can offer selectivity and sustainability. There are several reports on the transformation of *N*‐heteroaromatic compounds into *N*‐oxides both by whole‐cell biocatalysts (Kutanovas *et al*., 2013; Mitsukura *et al*., 2013; Stankevičiūtė *et al*., 2016) and application of purified enzymes (Ullrich *et al*., 2008). Most recently, metabolic engineering approaches that offer biosynthesis of specific pyrazine and phenazine *N*‐oxides using cellular components as starting materials have been introduced (Guo *et al*., 2020; Morgan and Li, 2020). Despite the recent progress, the application of biocatalytic methods is limited by the complicated synthesis procedure, biocatalyst’s inactivation and insufficient substrate scope. However, the main issues remain the low productivity, practicality and usability as none of the described biocatalytic approaches seriously challenges chemical oxidizers used in a laboratory‐scale production of ArN→O.

In this study, we describe a new whole‐cell biocatalysis system for ArN→O synthesis based on production of soluble di‐iron monooxygenase PmlABCDEF. By substituting *E. coli* host cells with certain *Pseudomonas* strains, we simplified the biotransformation procedure and vastly increased the productivity. Various pyridine, pyrazine and pyrimidine derivatives could be converted into appropriate *N*‐oxides on a preparative scale in the simple shake‐flask cultivation. This improved synthesis method was used to yield specific pyrazine and pyrimidine oxidation products that were difficult to obtain by employing a standard *m*CPBA‐based technique.

## Results

### Selecting a host for PmlABCDEF monooxygenase

Non‐heme iron‐dependent monooxygenases are multicomponent enzymes, which utilize dioxygen to catalyse a variety of different reactions including hydroxylation, epoxidation, sulphoxidation and require NAD(P)H as an electron donor (Leahy *et al*., 2003). They are known to be immensely challenging for heterologous protein biosynthesis, which often leads to relatively low activity in comparison to some other oxygenases, thus diminishing the potential biotechnological application (Torres Pazmiño *et al*., 2010). Despite our recent success in producing enzymatically active soluble di‐iron monooxygenase in *E. coli* and applying it for *N*‐oxidation purposes (Petkevičius *et al*., 2019), the issues concerning productivity have not been fully addressed. Although the formation of pyrazine *N*‐oxide was increased by employing the bioreactor fermentation, we were not able to boost the specific *N*‐oxidation activity in the simple shake‐flask biotransformation by any measures. Thus, we sought a microbial host that would be more suitable for the synthtesis of PmlABCDEF enzyme and the oxygenase‐based whole‐cell biocatalysis in general. *Pseudomonas* species feature a flexible redox metabolism, they show very high robustness against extreme environmental conditions or the presence of toxic substrates/products and inhibiting solvents and as a result, they have been intensively studied for potential application in industrial biotechnology (Poblete‐Castro *et al*., 2012). Our early attempts to produce PmlABCDEF monooxygenase in various *Pseudomonas* species identified two successful cases. One is *Pseudomonas putida* KT2440, a versatile microbe, well known for its use in whole‐cell biocatalysis (Poblete‐Castro *et al*., 2012). The other selected host, *Pseudomonas* sp. MIL9, came from our laboratory’s collection of microorganisms. Based on 16S rRNA phylogenetic analysis (Fig. S1), the closest relative species are *Pseudomonas umsongensis* and *Pseudomonas moorei* both of which are members of *P. jessenii* subgroup in genus *Pseudomonas* (Gomila *et al*., 2015). A particular interest in this microbe as a host was the presence of non‐heme di‐iron monooxygenase‐like genes (GenBank JAFEHE010000000, contig_00034). Apparently, due to a frameshift mutation, these genes do not translate into catalytically active enzyme (based on the inability of a wild type strain to perform any non‐heme di‐iron monooxygenase‐related transformations and a lack of the enzymatic activity of the recombinant proteins (data not shown). However, we speculated that the presence of such genetic material indicated the capability of *Pseudomonas* sp. MIL9 to provide an excellent cellular machinery for the biosynthesis of the heterologous non‐heme di‐iron monooxygenase such as PmlABCEDF. Thus, we opted to investigate and compare *E. coli* and both *Pseudomonas* biocatalysts for their performance in the production of aromatic *N*‐oxides.

### Biotransformation efficiency in different bacterial species

**Fig. 1:**
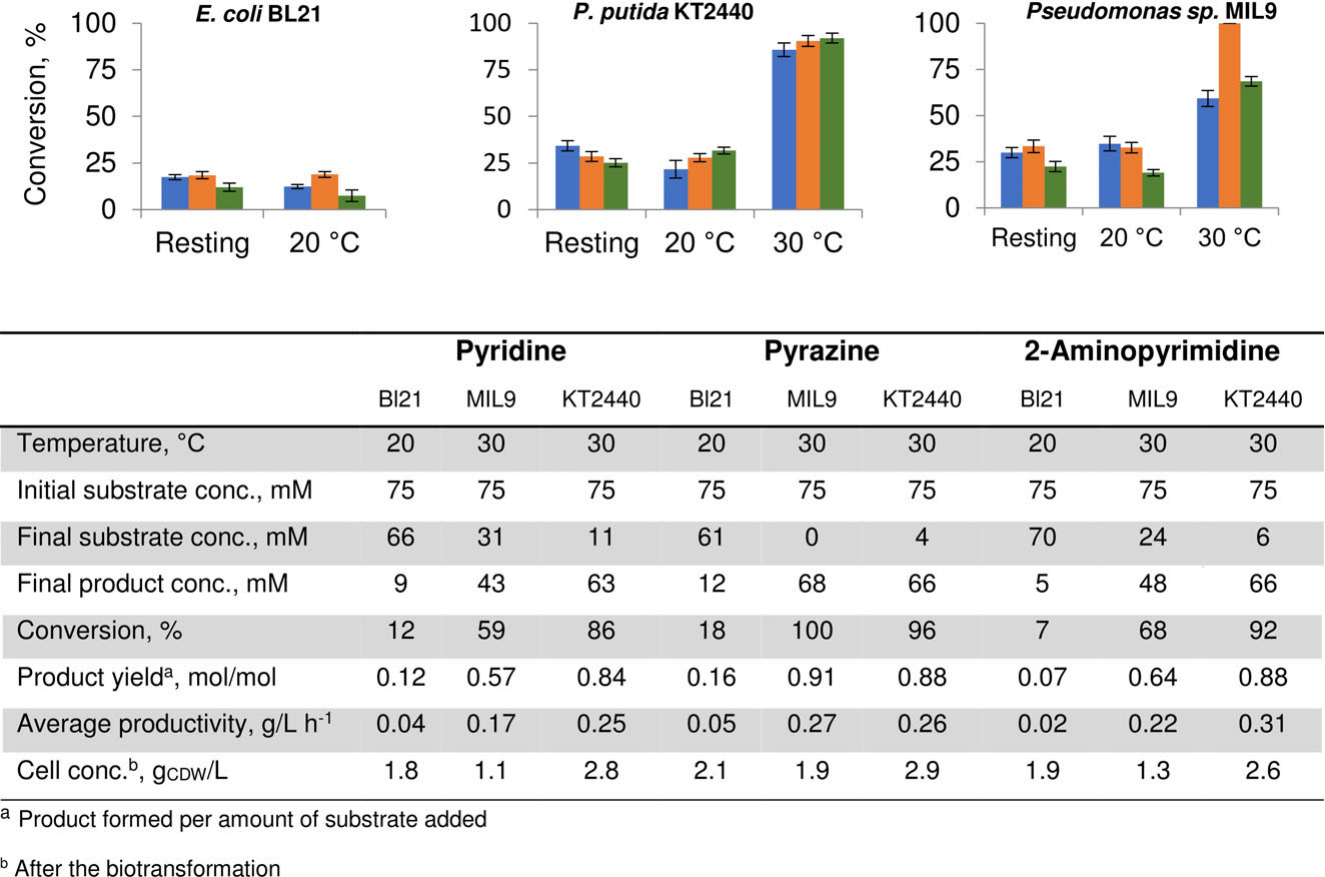
The performance of biotransformation in different biocatalysis systems after 24 h. The column diagram section shows the conversion of pyridine (blue), pyrazine (orange) and 2‐aminopyrimidine (green) by both resting cells and growing cells at 20 and 30°C, respectively. The table section displays a detailed biotransformation comparison between growing cells assays. In each case, a starting substrate concentration of 75 mM was used. The data represent mean values and standard deviations obtained from at least three independent cultivations.

The comparison of biotransformation effectiveness between strains was made under varying reaction conditions. The main differences occurred in plasmid vectors, which were used to express *pmlABCDEF*. *Pseudomonas* species harboured a hybrid, arabinose‐inducible vector, which was made by fusing regulatory elements of pBAD24 into the chassis of pBBR1MCS plasmid. Although this expression vector was tested in *E*. *coli* (*E. coli* bw25113), the performance of conversions did not match those of *E. coli* BL21 transformed with pET_pmlABCDEF plasmid; thus for further experiments, the later one was used for *E. coli* instead. Pyridine, pyrazine and 2‐aminopyrimidine were chosen as model substrates for the reason that they are the key building blocks of substrate scope described in this study. We compared the reaction parameters between resting and growing cells. Incubation temperature of 30°C was used for resting cells assay while growing cells biocatalysis was performed at 20 and 30°C, respectively. All strains were cultivated in 100 ml flat‐bottomed flasks with 20 ml of LB medium containing 1.0% of glucose. For a resting cell assay, a final concentration of 2 g_DCW_ l^‐1^ was used for all biocatalysts. Utilizing resting cells of *E. coli* as biocatalysts and an initial concentration of substrate of 75 mM, pyridine, pyrazine and 2‐aminopyrimidine were converted into pyridine‐1‐oxide (PNO), pyrazine‐1‐oxide (PyrazNO) and 2‐aminopyrimidine‐1‐oxide (2ANO) with an average conversion of 17%, 18% and 12%, respectively (Fig. 1). The increase in conversion was observed when *Pseudomonas* resting cells were used instead. Under these conditions, *P. putida* KT2440 strain was able to convert 34% of pyridine, 28% of pyrazine and 25% of 2‐aminopyrimidine. Quite similar results were obtained, when resting cells of *Pseudomonas* sp. MIL9 were employed. This biocatalyst transformed pyridine, pyrazine and 2‐aminopyrimidine into appropriate *N*‐oxides with conversion yields of 30%, 33% and 22%, respectively. Interestingly, growing cells at 20°C for both *E. coli* and *Pseudomonas* did not show any noteworthy increase or decrease in conversion effectiveness compared to analogous reactions performed with the resting cells at 30°C.

However, the critical point was reached performing biotransformations when growing cells were used at 30°C. The biosynthesis of PmlABCDEF in *E. coli* BL21 cells was shown to be susceptible to cultivation temperature as 20°C being optimal; induction at 30°C results in complete loss of *N*‐oxidation capacity. Unlike whole cells of *E. coli* BL21, both *Pseudomonas* strains, maintained catalytic activity at 30°C if the growing cells were used for biocatalysis. Moreover, the later approach resulted in the boost of a conversion degree. Growing cells of *P. putida* KT2440 on average converted 86% of pyridine, 96% of pyrazine and 92% of 2‐aminopyrimidine when an initial concentration of 75 mM of the substrate was used. Apparently, for this biocatalyst, the used concentration of substrates did not affect the cell growth (average cell concentration after 24 h of biotransformation was 2.8 g_CDW_ l^‐1^, 2.9 g_CDW_ l^‐1^ and 2.6 g_CDW_ l^‐1^ in the reaction broth with PNO, PyrazNO and 2ANO, respectively) as it was comparable to that without any substrate (2.8 g_CDW_ l^‐1^). On the other hand, whole cells of *Pseudomonas* sp. MIL9 completely transformed 75 mM of pyrazine to a corresponding pyrazin‐1‐oxide with traceable amounts of pyrazin‐1,4‐dioxide suggesting that even higher concentrations of pyrazine can be used. However, this biocatalyst seemed to be affected by high concentrations (75 mM) of pyridine and 2‐aminopyrimidine as the cell growth was weakened (average cell concentration after 24 h of biotransformation was 1.1 g_CDW_ l^‐1^ in the reaction broth with PNO and 1.3 g_CDW_ l^‐1^ with 2ANO) compared to pyrazine conversion (1.9 g_CDW_ l^‐1^) and growth without substrate (2.0 g_CDW_ l^‐1^) (Fig. 1). Nevertheless, 59% conversion of pyridine and 68% for 2‐aminopyrimidine were substantially higher than those achieved by resting cells or growing cells at 20°C. We opted to elucidate the best performance conditions for *N*‐oxidation using *Pseudomonas* growing cells producing PmlABCDEF.

### Optimization of biotransformation in *Pseudomonas* strains

**Fig. 2:**
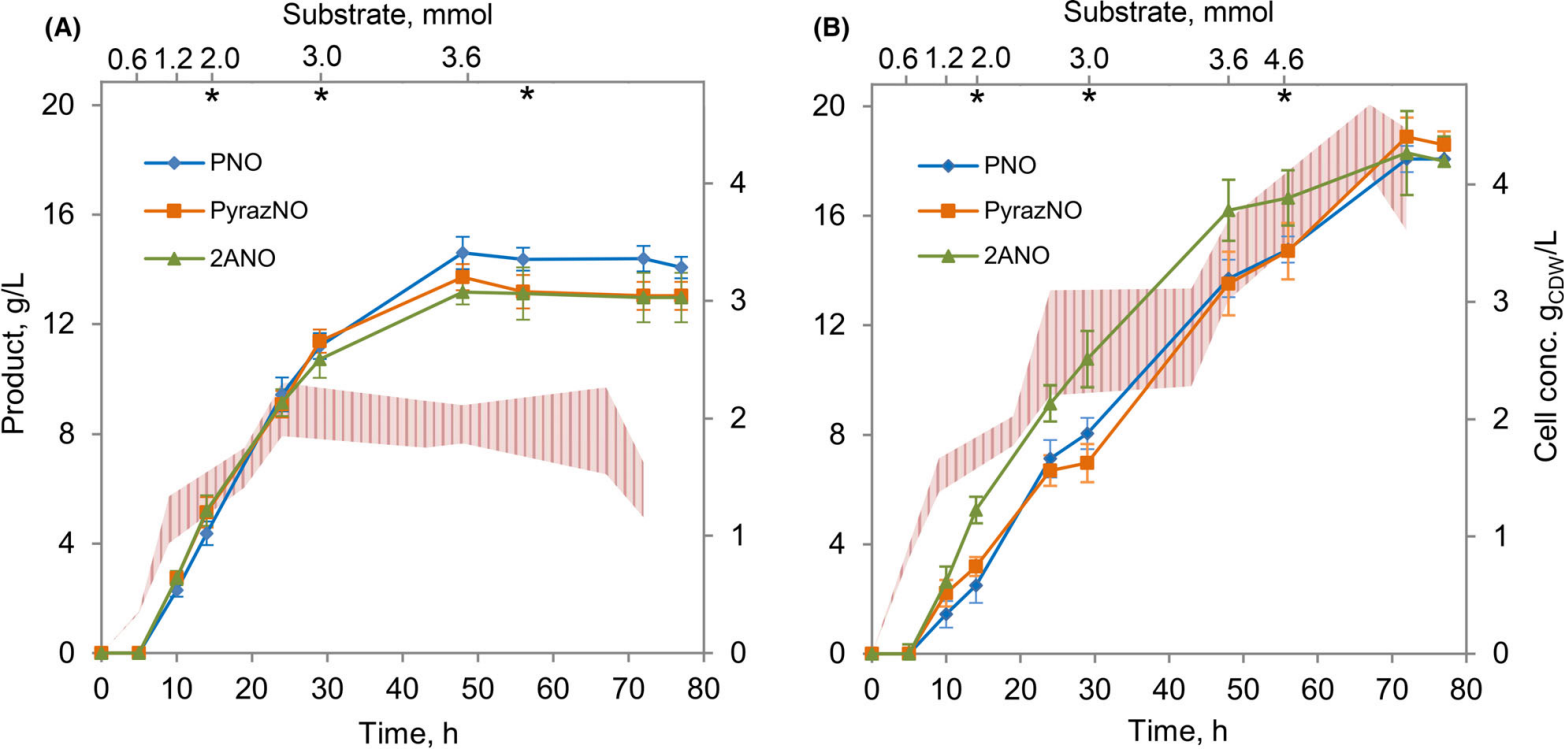
The comparison between biocatalysis using *Pseudomonas* sp. MIL9 (A) and *P. putida* KT2440 (B) growing cells. Curves present production of PNO (blue), PyrazNO (orange) and 2ANO (green). The cell growth with different substrates is represented by the plot area where the upper limit shows the highest average growth value while the lower limit displays the lowest average growth value from all three different transformations. The upper axis shows the total amount of substrate added into the reaction mixture on a time scale. Asterisks show the time point at which supplementation of glucose was carried out. The data represent mean values and standard deviations obtained from at least three independent cultivations.

The comparison of conversions using different strains suggested two key parameters for improvement: (i) duration of biotransformation and (ii) a proper way of substrate addition. A relatively high (75 mM) starting concentration of a substrate, in some cases, inhibited biomass growth that most likely affected the overall conversion. Also, unlike the system with *E. coli* BL21 cells, the duration of the reaction was not limited to 24 h using *Pseudomonas* cells as in both cases bacteria still featured catalytic activity. Moreover, the supplementation of the reaction broth with glucose to regenerate redox equivalents such as NADH seemed to have only a positive effect since it was able to improve cell growth as well as the performance of biotransformation. After a rough optimization, glucose was introduced in portions, starting with an initial concentration of 1.0% and a constant load of an additional 0.5% before every overnight cultivation (Fig. 2). Hence, ArN→O production was performed with growing *Pseudomonas* cells complying with their maintenance needs and substrate supplementation until the end of productive biotransformation. All reactions were performed in 100 ml flat‐bottomed flasks containing 20 ml of LB and 1.0% of glucose. Extra portions of glucose (250 µl of 40% solution) were added before every phase of overnight cultivation. Gene expression was initiated by adding 200 µl of 20% L‐arabinose solution at the beginning of the exponential growth (5 h from the start of the cultivation). This was followed by the supplementation of 0.6 mmol of an appropriate substrate, resulting in a final concentration of 30 mM. The progress of the bioconversion was monitored by TLC and HPLC‐MS to keep the track of substrate consumption as well as to maintain constant substrate feed. For the most part, supplementation of 0.6 mmol of the substrate was executed every 5–6 h, followed by the addition of 0.8–1.0 mmol for each phase of overnight cultivation. Biocatalysis using *Pseudomonas* sp. MIL9 cells reached their peak after 43 h of biotransformation (48 h of total cultivation) as no significant production increase was detected beyond this point (Fig. 2). The total amount of substrates added into the reaction reached 3.6 mmol, which would correspond to a total concentration of 180 mM. Pyridine exhibited the average conversion of 91%, pyrazine – 87% and 2‐aminopyrimidine – 72%. The determined final product concentrations were 157 mM for PNO, 141 mM for PyrazNO and 119 mM for 2ANO (Table 1). Apparently, in this assay, the presence of substrates or an accumulation of products did not influence the cell growth as average cell concentrations (2.0 g_CDW_ l^‐1^, 2.2 g_CDW_ l^‐1^ and 1.8 g_CDW_ l^‐1^ for reactions with PNO, PyrazNO and 2ANO, respectively) were similar to ones reached during cultivation without any substrate (2.1 g_CDW_ l^‐1^). On the other hand, whole cells of *P. putida* KT2440 demonstrated the catalytic activity for a longer period. The biotransformation was called complete after 67 h from the first substrate addition, as no substantial product formation was detected afterwards. During this period, a total of 4.6 mmol of each substrate were added, corresponding to an end concentration of 230 mM. The average conversion of 87% was observed for pyridine, 98% for pyrazine and 79% for 2‐aminopyrimidine. After measuring the final product concentrations, we found out 189 mM of PNO, 199 mM of PyrazNO and 165 mM of 2ANO. During this type of biotransformation, there was no significant impact on the growth of *P. putida* KT2440 strain regarding the accumulation of reaction products. The cultivation of *P. putida* KT2440 under analogous conditions only without any addition of a substrate resulted in an average cell concentration of 4.8 g_CDW_ l^‐1^, which was similar to cultivations when *N*‐oxides were produced (4.7 g_CDW_ l^‐1^ for PNO, 4.4 g_CDW_ l^‐1^ for PyrazNO and 4.1 g_CDW_ l^‐1^ for 2ANO). Because of the longer biotransformation time, utilizing *P. putida* KT2440 whole cells resulted in greater product titres, though *Pseudomonas* sp. MIL9 biocatalyst featured slightly higher average productivity (Table 1). The clear differences between these biocatalysts regarding conversion performances appeared in the first phase (0–30 h) of the biotransformation. During this period, *P. putida* KT2440 cells exhibited *N*‐oxide production rates of 0.28 g l^‐1^ h^–1^ for PNO, 0.24 g l^‐1^ h^–1^ for PyrazNO and 0.31 g l^‐1^ h^–1^ for 2ANO, and they were similar to the average productivity of the complete conversion. However, *Pseudomonas* sp. MIL9 strain displayed productivities of 0.39 g l^‐1^ h^–1^ for PNO, 0.39 g l^‐1^ h^–1^ for PyrazNO and 0.37 g l^‐1^ h^–1^ for 2ANO (Fig. 2). Interestingly, it seemed that the catalytic activity was maintained mainly during the exponential growth and begun to reach a plateau through the stationary phase. It seems that a bio‐oxidation capacity closely correlates with the growth phase in which cells stay metabolically active and are capable of the regenerating of reducing equivalents such as NADH since the addition of glucose at the stationary phase did not enhance the conversion.

**Table 1:** The comparison of biotransformation parameters between *Pseudomonas sp*. MIL9 and *P. putida* KT2440 growing cells. The data represent average values obtained from at least three independent cultivations.

	*Pseudomonas* sp. MIL9			*P. putida* KT2440		
	Pyridine	Pyrazine	2‐Aminopyrimidine	Pyridine	Pyrazine	2‐Aminopyrimidine
Duration of biotransformation, h	43	43	43	67	67	67
Total substrate conc.^a^, mM	180	180	180	230	230	230
Final substrate conc., mM	17	23	51	29	4	47
Final product conc., mM	157	141	119	189	199	165
Conversion, %	91	87	72	87	98	79
Product yield^b^, mol/mol	0.87	0.78	0.66	0.82	0.87	0.72
Average productivity, g l^‐1^ h^‐1^	0.35	0.31	0.31	0.27	0.29	0.27
Cell conc.^c^, g_CDW_ l^‐1^	2.0	2.2	1.8	4.7	4.4	4.1
Total product titre, g l^‐1^	14.9	13.5	13.2	18.0	19.1	18.3

^a^ Theoretical concentration of summing all supplementations of a substrate.

^b^ Product formed per amount of substrate added.

^c^ After the biotransformation.

### Biocatalytic synthesis of ArN→O using *Pseudomonas* strains

The differences between *Pseudomonas* strains as biocatalysts were exploited to maximize the effectiveness of the ArN→O synthesis. For the most bio‐convertible substrates without apparent inhibition of cell growth and possessing relatively high solubility in water, *Pseudomonas* sp. MIL9 whole cells were chosen as primary biocatalyst since they offered a reduced conversion time compared to *P. putida* KT2440. Without aforementioned pyridine (**1a**), pyrazine (**2a**) and 2‐aminopyrimidine (**3a**), these substrates also included 2‐amino‐4‐methylpyridine (**4a**), 2‐amino‐4‐methylpyridine (**5a**) and 2‐methylpyrazine (**6a**) (Table 2). All of those compounds underwent a full conversion in 43 h or less. During this time, *Pseudomonas* sp. MIL9 cells were able to fully transform at least 5 mmol of each compound in 50 ml of the reaction medium. This would roughly correspond to the final product concentration of 100 mM and higher. Another group of substrates composed of 2‐amino‐4‐chloropyrimidine (**7a**), 2‐amino‐4‐chloropyridine (**8a**), 2‐amino‐4,6‐dimethylpyrimidine (**9a**), 2‐amino‐4‐bromopyridine (**10a**), 2‐chloropyrazine (**11a**), 2‐amino‐4‐methoxypyrimidine (**12a**), 4‐aminopyrimidine, pyrazine‐2‐carbonitrile (**13a**) and 4‐methylpyrimidine (**14a**). These compounds in a total amount of 2.5–5 mmol in 50 ml of the reaction mixture were fully converted that would correspond to 50–100 mM of product concentration. Apparently, in this case, there was no big difference in which biocatalyst to use as both of them performed similarly. However, the most resistant substrates to PmlABCDEF needed a prolonged exposure to reach the full conversion; thus in this instant, the use of *P. putida* KT2440 growing cells was a better option. Such compounds were 2‐amino‐4‐methyl‐3‐nitropyridine (**15a**), 4‐amino‐2‐methoxypyrimidine (**16a**), 2‐amino‐3‐nitropyridine (**17a**) and 2‐aminopyridine‐3‐carbonitrile (**18a**). The full conversion was achieved using 1–2.5 mmol of a substrate, yielding an assumed product concentration of 20–50 mM in 50 ml of the reaction broth.

**Table 2:** List of substrates that were set for bio‐oxidation and product synthesis. Compounds were allocated based on biocatalyst choice and productivity (range of potential product concentration is shown in the brackets).

*Pseudomonas* sp. MIL9 (> 100 mM)		*Pseudomonas* sp. MIL9 or *P. putida* KT2440 (50–100 mM)		*P. putida* KT2440 (20–50 mM)	
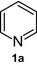	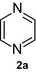	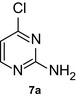	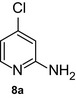	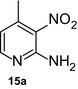	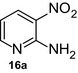
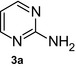	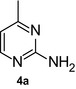	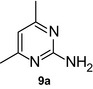	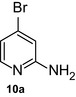	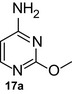	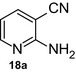
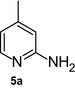	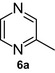	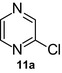	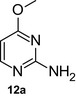		
		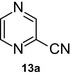	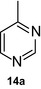		

### Biocatalysis versus chemical oxidation

**Fig. 3:**
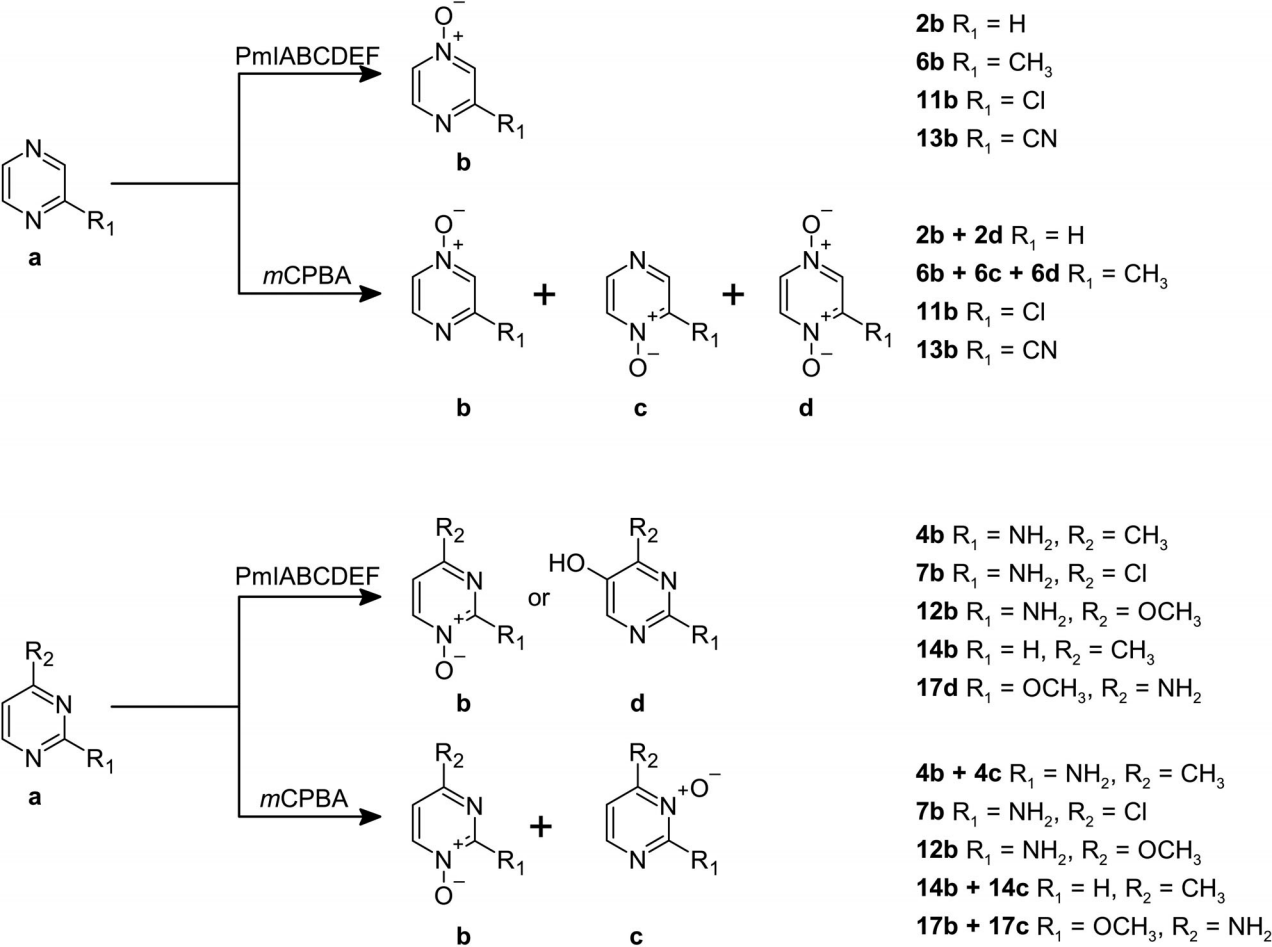
Representation of the synthesis pathways for pyrazine and pyrimidine *N*‐oxides utilizing different catalysts. The upper arrow presents biocatalysis method based on production of soluble di‐iron monooxygenase PmlABCDEF in *Pseudomonas* strains, while the lower arrow displays oxidation using *m*CPBA.

Once we established the biocatalysis system for preparative scale ArN→O synthesis, we aimed to challenge *m*CPBA‐based oxidation as this is the most typical chemical reagent used for the laboratory‐scale synthesis of ArN→O (Vörös *et al*., 2014). For some pyrazine derivatives, *N*‐oxidation with *m*CPBA results in unwelcome side reactions including the formation of di‐*N*‐oxides or isomeric mono‐*N*‐oxides as in the case of asymmetric pyrazines bearing various alkyl substituents (Sato, 1985; Butler and Cabrera, 2013). However, di‐*N*‐oxide formation from compounds **2a** and **6a** was easily handled in the case of a biocatalytic method (Fig. 3). A simple monitoring of the biotransformation was enough as di‐*N*‐oxides started to form after the prolonged exposure when a full conversion of the initial substrate has already been achieved. It seemed that to some degree, PmlABCDEF‐based oxidation was able to deal with regioselectivity issues as well. Asymmetric pyrazine derivatives **6a**, **11a** and **13a** were converted by a biocatalytic *N*‐oxidation into single products **6b**, **11b** and **13b**, respectively, all of which bear an oxygen moiety at the less hindered nitrogen. For compounds **11a** and **13a**, this must be driven more by the chemical nature (deactivating lone‐pair electrons of nitrogen by *ortho* electron‐withdrawing substituents) rather than enzymatic preference. Nevertheless, the biocatalytic oxidation of **6a** was an explicit example of regioselective synthesis as only mono‐*N*‐oxide **6b** was produced. Together with the previously described production of 3,5‐dimethylpyrazine 1‐oxide and 2,3,5‐trimethylpyrazine 1‐oxide (Petkevičius *et al*., 2019), this type of biocatalysis makes a strong case for the selective synthesis of asymmetric alkylpyrazine *N*‐oxides.

Various monosubstituted pyrimidines and methylpyrimidines, in general, are very susceptible to side reactions (decomposition, annular carbon oxidation, ring‐opening) accompanying *N*‐oxidation in the presence of different peracids (Jovanovic, 1984). Moreover, unsymmetrical pyrimidines can potentially yield two isomeric compounds making all of these compounds very challenging to oxidize selectively. In this study, the substrates **7a** and **12a** were transformed to the appropriate single products employing both synthesis methods, biocatalysis and *m*CPBA oxidation. Upon close investigation of literature (Jovanovic, 1984) and NMR data, those products were identified as **7b** and **12b,** respectively; an attack to less hindered nitrogen atom was observed. This was also supported by our previous investigation where it was shown that particular features of undesirable substrates for PmlABCDEF included two *ortho*‐substituents. Unlike chemical oxidation, a biocatalytic conversion of compounds **4a**, **14a** and **17a** produced only a single product in each sample. As expected, compounds **4a** and **14a** were transformed into *N*‐oxides **4b** and **14b** bearing oxygen moiety at the N‐1 position. Interestingly, HPLC‐MS data of the bio‐oxidation product of **17a** did not match those of **17b** and **17c** that were produced by chemical oxidation. The analysis of NMR data revealed the formation of compound **17d** (4‐amino‐2‐methoxypyrimidin‐5‐ol), a case where an expected *N*‐oxidation shifted to a ring hydroxylation at C‐5 position instead. Although it is an exceptional case in PmlABCDEF‐based biocatalysis, this is a noteworthy example for further studies as a selective ring hydroxylation in heteroaromatic compounds is a very difficult reaction to achieve using the known methods of chemical synthesis.

## Discussion

The results of this study not only provide important data on the biocatalytic synthesis of ArN→O but also might share some insights on the biotechnological application of the non‐heme di‐iron monooxygenases in general. The ability of these biocatalysts to oxidize a variety of different compounds for biocatalytic applications has been widely studied, though little progress has been made to produce them in various recombinant hosts such as *E. coli* or *Pseudomonas putida* (Torres Pazmiño *et al*., 2010). Thus far, monooxygenases such as soluble methane monooxygenase (sMMO) of *Methylosinus trichosporium* OB3b (Sullivan *et al*., 1998) and propene monooxygenase from *Mycobacterium* sp. strain M156 (Chan Kwo Chion *et al*., 2005) have been successfully produced in the parental strains deficient in the appropriate monooxygenase. Others non‐heme di‐iron monooxygenases, like toluene *o*‐xylene monooxygenase (TOM) from *Burkholderia cepacia* G4, toluene/benzene 2‐monoxygenase of *Burkholderia* sp. strain JS150, toluene *o*‐xylene monooxygenase (ToMO) of *Pseudomonas stutzeri* OX1, toluene *para*‐monooxygenase (T3MO) of *Ralstonia pickettii* PKO1, toluene 4‐monooxygenase (T4MO) from *Pseudomonas mendocina* KR1 and phenol hydroxylase (PH) of *Pseudomonas stutzeri* OX1 have been known for the successful heterologous biosynthesis (Nichol *et al*., 2015). However, the aforementioned studies were focussed on investigating genetic or enzymatic properties rather than the application for the synthesis of targeted compounds due to the low productivity of biocatalytic systems. This has been an indication that there are additional host factors that are vital for optimal monooxygenase function, and it was demonstrated in the study of three‐component alkane monooxygenase (AlkB) of *Pseudomonas oleovorans* GPo1. It was shown that despite considerable production of alkane monooxygenase in some *E. coli* hosts, the most active recombinants showed *in vivo* alkane‐oxidation rates no higher than that of the native host strain (Staijen *et al*., 2000). Authors suggested that the intracellular environment (protein misfolding and incorrect processing, incomplete iron incorporation, the improper ratio of enzyme components) places significant and rather specific restrictions on the synthesis, stability and activity. The importance of host selection was also demonstrated in the recent study of di‐iron monooxygenases, where a three‐component monooxygenase from *Rhodococcus wratislaviensis* was produced as an active form in recombinant *Rhodococcus erythropolis* cells and applied for the synthesis of α‐methyl‐D‐serine (Hibi *et al*., 2021). In our case, the heterologous production of PmlABCDEF in *E. coli* resulted in the biosynthesis of active form only at 20°C or lower, while induction at 30°C produced insoluble proteins and exhibited no catalytic activity (Fig. S2). On the contrary, biosynthesis of PmlABCDEF as well as catalytic activity was favoured at 30°C in *Pseudomonas* hosts. We should keep in mind that PmlABCDEF shares high similarity to counterparts of *Pseudomonas* genus as it shows overall ˜ 90% amino acid sequence identity to YHS domain‐containing proteins as well as putative monooxygenases of various *Pseudomonas* species implying *pmlABCDEF* originated from this genus. Thus, we can make an educated guess that re‐introducing *pmlABCDEF* genes into specific *Pseudomonas* hosts provided a suitable intracellular environment and enabled us to achieve stable and efficient protein biosynthesis. Also, unlike the metabolism of glucose in *E. coli*, *Pseudomonas* strains are known to employ the Entner‐Doudoroff pathway, the Embden‐Meyerhof‐Parnas pathway and the pentose phosphate pathway for the metabolism of glucose (Nikel *et al*., 2015). Ultimately, this biochemical network favours the processing of biochemical resources towards the production of NAD(P)H rather than the generation of ATP (Martínez‐García and de Lorenzo, 2019). The elevated levels of reductive equivalents make *Pseudomonas* species a phenomenal host for redox‐intensive reactions such as PmlABCDEF‐based *N*‐oxidation.

Interestingly, the whole‐cell systems, including those exploiting non‐heme di‐iron monooxygenases, have been attributed to ArN→O production, which helps to put into perspective the capabilities of the presented synthesis method. For instance, oxidation of pyridine to pyridine *N*‐oxide has been demonstrated employing phenol‐degrading bacteria *Diaphorobacter* sp. J5‐51, *Acinetobacter* sp. SJ‐15, *Acinetobacter* sp. SJ‐16, *Acidovorax* sp. J5‐66 and *Corynebacterium* sp. JOR‐20 as this activity was assigned to the presence of phenol monooxygenases (Sun *et al*., 2014). After 70 h of cultivation under optimum conditions, 100 mg l^‐1^ (1.3 mM) of pyridine was completely transformed. Additionally, phenol hydroxylase gene with six components *pheKLMNOP* was heterologously expressed in the non‐pyridine‐degrading *Pseudomonas* sp. CO‐44 that resulted in detection of pyridine *N*‐oxide, though any noteworthy boost of productivity has not been stressed out. The capability of *Burkholderia* sp. MAK1 whole cells to oxyfunctionalize a variety of pyridine derivatives, including several *N*‐heterocyclic ring systems to corresponding *N*‐oxides, was linked to the activity of atypical non‐heme di‐iron monooxygenase HpdABCDE (Petkevičius *et al*., 2018). The induced resting cells possessed a capacity to consume up to 0.5–1.0 mM concentration of a substrate in 100 ml reaction buffer over 24–48 h (Stankevičiūtė *et al*., 2016). However, the expression of monooxygenase gene in *hpdABCDE*‐minus mutant did not significantly improve catalytic capabilities. More advanced biocatalysis systems for ArN→O synthesis include *Verticillium* sp. GF39 fungi cells (Mitsukura *et al*., 2013), *Rhodococcus jostii* TMP1 whole cells (Kutanovas *et al*., 2013) and artificial metabolic pathway in *Pseudomonas chlororaphis* HT66 (Guo *et al*., 2020). The later approach produced 1‐hydroxyphenazine *N*′10‐oxide in product titre of 143.4 mg l^‐1^, *Verticillium* sp. GF39 formed 5 mM 1‐methylisoquinoline *N*‐oxide from 1‐methylisoquinoline with a molar conversion yield of 100% after a 10 h incubation at 20°C and *Rhodococcus jostii* TMP1 apparently was able to catalyse *N*‐oxidation of 2,3‐dimethylpyrazine and 3‐ethylpyridine utilizing 8–10 mM concentration of a substrate in 96 h. Keeping all that in mind, the application of PmlABCEDF monooxygenase stands out as an exceptional biocatalysis system for producing ArN→O. Not only it features a simplified procedure and broad substrate spectrum, the recent upgrade improved the productivity up to the level that it could rival chemical synthesis methods.

## Conclusions and future perspectives

The results presented in this study show that production of PmlABCDEF monooxygenase in *Pseudomonas putida* KT2440 and *Pseudomonas* sp. MIL9 strains serves as a powerful bio‐oxidation catalyst. The use of two different biocatalysis systems enabled diverse synthesis approaches for aromatic *N*‐oxides. *Pseudomonas* sp. MIL9 was preferred for quick and productive biotransformation, while *Pseudomonas putida* KT2440 was used in case maximum production of the targeted compound was needed. The later strain is a well‐described biocatalyst; thus, additional improvements for ArN→O production, including metabolic engineering and gene editing, are possible. It seems that *Pseudomonas* sp. MIL9 strain is also a promising host for all sorts of biotransformation. Most recently, the genome analysis of the closely relative strain *Pseudomonas umsongensis* GO16 has shown the metabolic versatility and the potential for biotransformations (Narancic *et al*., 2021). Since the genome sequencing data of *Pseudomonas* sp. MIL9 are also available (GenBank: JAFEHE010000000); further investigations, including the comparison and analysis of genetic data, should help to develop new biotransformation platforms.

The recombinant *Pseudomonas* strains were able to catalyse *N*‐oxidation of various pyridine, pyrazine and pyrimidines with high conversion and product yield. Compared with known biocatalysis techniques for aromatic *N*‐oxide production, the presented approach emerges as the most productive biotransformation method by far. The achieved level of productivity allows using of this method for practical application on the laboratory‐scale level and in some cases to replace a more common *m*CPBA‐based oxidation if a certain regioselective synthesis is required. The development of a suitable biocatalysis chassis allows key decisions on the design of this biotransformation method to be made more rapid and effective under conditions that are closer to industrial settings. Similar to other microbial‐based industrial processes, a transition from a test tube reaction to a small bioreactor frequently encounters serious obstacles. In this case, a big challenge lays underneath the difficulty of downstream processing for recovering synthesized *N*‐oxides of interest. A possible solution may be a proper two‐phase liquid–liquid cultivation system with an aqueous and an organic phase. In recent years, such new reactor configurations have been developed making downstream processing easy as well as dealing with toxicity effects (Verhoef *et al*., 2009). Also, a new biotransformation matrix should implement changes in the reaction medium, shifting from undefined nutrient broth to defined mineral medium. There are a variety of different defined media used for *Pseudomonas* cultivation, including a recent approach where high cell density (CDW of 102 g l^–1^) cultivation of *Pseudomonas putida* KT2440 was reached even without a supply of oxygen‐enriched air (Davis *et al*., 2015). Integration of such growth strategy into existing or currently developed pipelines of utilizing renewable feedstocks or industrial wastes has the potential for the development of a sustainable bioproduction of desired *N*‐oxides. The presented biocatalysis system is an exceptional example of non‐heme di‐iron monooxygenases application, which are not limited to *N*‐oxidation reactions and other synthesis routes including hydroxylation, sulphoxidation and epoxidation should follow. Together with a naturally high tolerance of *Pseudomonas* species to extreme and toxic conditions as well as flexible genetic modifications, these microbial cell factories have an excellent starting position for further development of them into the effective production platforms for various chemicals, which are not accessible so far.

## Experimental procedures

### Materials

Pyridine, 2‐aminopyrimidine, 2‐aminopyridine‐3‐carbonitrile, 2‐amino‐4‐methyl‐3‐nitropyridine, pyrazine and pyrazine‐2‐carbonitrile were obtained from Sigma‐Aldrich (Munich, Germany), and 2‐amino‐4‐chloropyridine, 2‐amino‐4‐bromopyridine and 2‐amino‐4‐methylpyridine were products of Combi Blocks Inc (San Diego, CA, USA). 2‐Amino‐4‐methoxypyrimidine, 4‐amino‐2‐methoxypyrimidine, 2‐amino‐4‐methylpyrimidine, 2‐chloropyrazine, 2‐methylpyrazine and 4‐methylpyrimidine were purchased from Apollo Scientific (Bredbury, UK), when 2‐amino‐4,6‐dimethylpyrimidine was ordered from TCI EUROPE N.V. (Belgium), and 2‐amino‐3‐nitropyridine was bought from Merck (Darmstadt, Germany). *meta*‐Chloroperoxybenzoic acid was purchased from Alfa Aesar (Ward Hill, MA, USA). Chloroform, methanol and acetonitrile were products from Firma Chempur (Piekary Śląskie, Poland).

### Bacterial strains and growth conditions

The bacterial strains used in this study are listed in Table 3. *E. coli* DH5α (Thermo Fischer Scientific, Vilnius, Lithuania) was used as a host for gene cloning and plasmid isolation. *E. coli* BL21 (DE3), *Pseudomonas putida* KT2440 and *Pseudomonas* sp. MIL9 were employed as hosts for recombinant protein production. All bacterial strains were routinely grown in Luria‐Bertani (LB) broth supplemented with kanamycin (100 μg ml^‐1^) if necessary. Composition of media was as follows: LB (g l^‐1^) – tryptone (Formedium, Hunstanton, UK) 10.0, yeast extract (Merck, Darmstadt, Germany) 5.0, NaCl (Fluka, Buchs, Switzerland) 5.0; SOB (g l^‐1^) – tryptone (Formedium) 20.0, yeast extract (Merck) 5.0, NaCl (Fluka) 0.5, KCl (Merck) 0.2, MgCl_2_·6H_2_O (Reachim, Moscow, Russia) 2.0, MgSO_4_·7H_2_O (Reachim) 2.5. Before sterilization at 1 atm for 30 min. pH adjustment to 7.2 was made. Strains of *E. coli* were grown at 30–37°C temperature, while *Pseudomonas* strains were cultivated at 30°C.

**Table 3:** Plasmids and strains used in this study.

Plasmid or strain	Relevant characteristics	Source or reference
pET_pmlABCDEF	Recombinant pET‐28b containing *pmlABCDEF* gene	Petkevičius *et al*. (2019)
pBAD2‐MCS‐1	The amplicon containing regulatory elements of pBAD24 fused into the chassis of pBBR1MCS	Petkevičius *et al*. (2018)
Pml_pBAD2	Recombinant pBAD2‐MCS‐1 containing *pmlABCDEF* gene	This study
Strain		
*E. coli* DH5α	F^–^ *endA1 glnV44 thi‐1 recA1 relA1 gyrA96 deoR nupG purB20* φ80d*lacZ*ΔM15 Δ(*lacZYA‐argF*)U169, hsdR17(*r_K_* ^–^ *m_K_* ^+^), λ^–^	Thermo Fischer Scientific, Lithuania
*E. coli* BL21 (DE3)	F^–^ *ompT gal dcm lon hsdS_B_* (*r_B_* ^–^ *m_B_* ^–^) λ(DE3 [*lacI lacUV5*‐*T7p07 ind1 sam7 nin5*]) [*malB* ^+^]_K‐12_(λ^S^)	Novagen, Germany
*Pseudomonas putida* KT2440	Plasmid‐free derivative of a toluene‐degrading bacterium strain *Pseudomonas putida* mt‐2	DSM 6125 DSMZ, Germany
*Pseudomonas* sp. MIL9	Isolated from soil; Malkų Bay, Klaipėda District Municipality, Lithuania	This study

### Transformation of bacterial strains

The introduction of plasmid DNA into *E. coli* strains was carried out *via* heat shock. Standard procedures and techniques were used for the transformation and the preparation of chemically competent cells. The transformation of *Pseudomonas* strains was executed according to the modified protocol of Cho *et al*. (1995). The portion of overnight culture (0.5 ml) was transferred into 50 ml of fresh LB medium and was grown with shaking at 30°C until the early log phase (OD_600_ 0.3–0.5). The cells were harvested by centrifugation at 4000 g for 10 min. at 4°C, washed with 5 ml of ice‐cold glycerol solution (10%) and re‐centrifuged. This washing procedure was repeated two more times, and finally, cells were suspended in 0.5 ml of ice‐cold glycerol solution (10%). The resulting mixture was divided into five aliquots of 100 μl that were immediately used for transformation, unlike *E. coli*, *Pseudomonas* cells (mostly for the MIL9 strain) could not be electroporated with high efficiency after being frozen. 100 μl of cell suspension was mixed with 5 μl of plasmid DNA (˜100 ng μl^‐1^) and transferred to chilled 0.2 cm gap cuvettes. Following the delivery of the pulse (12 kV cm^‐1^, a time constant ˜ 5 ms), the cells were mixed with 0.9 ml of SOB medium and shaken for 1 h at 30°C before transferring onto LB‐agar plates containing appropriate antibiotic (cultivated at 30°C overnight).

### Biotransformation conditions for whole‐cell biocatalysis

The detailed description of conversion settings using *E. coli* BL21 (DE3) as a host was outlined in our previous study (Petkevičius *et al*., 2019). The appropriate *Pseudomonas* strain (KT2440 or MIL9, respectively) transformed with Pml_pBAD2 plasmid was grown overnight in 10 ml of LB medium supplemented with kanamycin (final concentration 40 μg ml^‐1^) and glucose (final concentration 1.0 g ml^‐1^). The portion of overnight culture (0.5 ml) was transferred into 50 ml of fresh LB medium supplemented with kanamycin (final concentration 40 μg ml^‐1^) and glucose (final concentration 1.0 g ml^‐1^). The incubation was executed with shaking (200 rpm) at 30°C until the early log phase (OD_600_ 0.3–0.5), and then, 0.5 ml of 20% arabinose solution was added. This was followed by the addition of 0.5–1.5 mmol of the appropriate substrate, resulting in starting substrate concentration of 10–30 mM. Depending on the bio‐convertibility of the used substrate, 1 to 4 additional portions of 0.5–1.5 mmol were introduced into the reaction mixture during the course of the complete biotransformation. Before every phase of overnight cultivation, 625 µl of 40% glucose solution was added to the cultivation broth. *Pseudomonas* strains were also tested with each substrate for the possible unspecific activity or intrinsic oxidation capabilities as samples with appropriate cells without Pml_pBAD2 plasmid were prepared (no such activity was found for substrates used in this study).

### Isolation of the reaction products

The reaction mixture was separated from biomass by centrifugation (4000 g for 30 min). The volume of the supernatant was reduced under vacuum to a volume of 5–10 ml, and it was transferred to the separation funnel. Then, water phase was washed with 20–40 ml of chloroform at least three‐four times. The organic phases were combined and dried with anhydrous Na*_2_* SO_4_. The resulting solution was evaporated under reduced pressure to give a crude product. Additionally, a purification procedure by silica gel flash chromatography with CHCl_3_‐MeOH (5:1) could be applied to obtain a refined product. However, this purification step was mandatory on two occasions: (i) when a biotransformation product could not be extracted due to poor solubility in the organic phase, the supernatant of the conversion mixture was dried out under vacuum and gently resuspended in a mixture of chloroform and methanol and transferred onto chromatography column; (ii) the anticipated *N*‐oxide was synthesized employing *m*CPBA. All isolation stages were monitored by TLC. The purity of the final product was verified by high‐performance liquid chromatography–mass spectrometry (HPLC‐MS) and the chemical structure was confirmed by nuclear magnetic resonance (NMR) spectroscopy.

### HPLC‐MS analysis

The sample (0.5 ml) of the whole‐cell biocatalysis reaction was transferred to a 1.5 ml tube and mixed with an equal part of acetonitrile. After the mixture was centrifuged (12 000 g for 5 min), 0.5 ml of supernatant was analysed using a high‐performance liquid chromatography system. HPLC‐MS analysis was performed using a high‐performance liquid chromatography system (Shimadzu, Japan) equipped with a photo diode array (PDA) detector and a mass spectrometer (LCMS‐2020; Shimadzu) equipped with an ESI source. The data were analysed using the LabSolutions LCMS software.

### ^1^H NMR and ^13^C NMR

NMR spectra were recorded in DMSO‐d_6_ or CDCl_3_ on an Ascend 400: ^1^H NMR – 400 MHz, ^13^C NMR – 101 MHz (Bruker, MA, USA). Chemical shifts (δ) are reported in ppm relative to the solvent resonance signal as an internal standard.

### Analytics

Conversion is expressed as ((I_a_ – F_a_)/I_a_) × 100%, where I_a_ – the initial amount of a substrate before the conversion, F_a_ – the final amount of a substrate after conversion. The substrate’s amount was determined by integrating the absorbance area of a particular peak in the HPLC chromatogram. If no substrate was left, the conversion is defined as complete (> 99%). Volumetric productivity was defined as the amount of product (g) produced per volume (l) of the reaction per time (h) under optimum conditions. Product yield is expressed as P_t_ (mol)/ P_f_ (mol), where P_t_ – theoretical amount of product formed based on the degree of conversion; P_f_ – the amount of product found after the biotransformation. Cell dry weight (CDW) was determined after biotransformation was completed. A titre of the reaction was described as the total amount (g) of product formed in a conversion mixture (l) during the whole‐cell biocatalysis.

### Genome sequencing and analysis

This Whole Genome Shotgun project has been deposited at DDBJ/ENA/GenBank under the accession JAFEHE000000000. The version described in this paper is version JAFEHE010000000.

## Funding Information

This project has received funding from European Social Fund No 09.3.3‐LMT‐K‐712 ‘Development of Competences of Scientists, other Researchers and Students through Practical Research Activities’ under a grant agreement with the Research Council of Lithuania (LMTLT).

## Conflict of interest

None declared.

## Supporting information


**Fig. S1**. Maximum‐likelihood phylogenetic tree based on the partial 16S rRNA gene sequences of members of the *Pseudomonas* genus. The percentage of 500 trees in which the associated taxa clustered together after a bootstrap analysis is shown next to the branches. The 16S rRNA sequence of *E. coli* K12 was used as an outgroup.

**Fig. S2**. PmlABCDEF biosynthesis analysis in different hosts. Calculated molecular weight of individual PmlABCDEF subunits: PmlA – 11 kDa, PmlB – 38 kDa, PmlC – 10 kDa, PmlD – 59 kDa, PmlE – 13 kDa, PmlF – 39 kDa. Negative controls (C_N_) is a cell‐free extract of *E. coli* BL‐21 and *Pseudomonas* sp. MIL9 respectively. (T) indicates a total fraction of cell‐free extract, (S) – soluble fraction.

## References

[mbt213849-bib-0001] Butler, M. , and Cabrera, G.M. (2013) Determination of the position of the N‐O function in substituted pyrazine N‐oxides by chemometric analysis of carbon‐13 nuclear magnetic resonance data. J Mol Struct 1043: 37–42.

[mbt213849-bib-0002] Chan Kwo Chion, C.K. , Askew, S.E. , and Leak, D.J. (2005) Cloning, expression, and site‐directed mutagenesis of the propene monooxygenase genes from *Mycobacterium* sp. strain M156. Appl Environ Microbiol 71: 1909–1914. 15812019 10.1128/AEM.71.4.1909-1914.2005 PMC1082536

[mbt213849-bib-0003] Cho, J.‐H. , Kim, E.‐K. , and So, J.‐S. (1995) Improved transformation of *Pseudomonas putida* KT2440 by electroporation. Biotechnol Tech 9: 41–44.

[mbt213849-bib-0004] Chucholowski, A.W. , and Uhlendorf, S. (1990) Base catalyzed rearrangement of 5‐cyanomethyl‐2‐isoxazolines; novel pathway for the formation of 2‐aminopyridine N‐oxides. Tetrahedron Lett 31: 1949–1952.

[mbt213849-bib-0005] Davis, R. , Duane, G. , Kenny, S.T. , Cerrone, F. , Guzik, M.W. , Babu, R.P. , Casey, E. , and O'Connor, K.E. (2015) High cell density cultivation of *Pseudomonas putida* KT2440 using glucose without the need for oxygen enriched air supply. Biotechnol Bioeng 112: 725–733. 25311981 10.1002/bit.25474

[mbt213849-bib-0006] Dyer, R.M.B. , Hahn, P.L. , and Hilinski, M.K. (2018) Selective heteroaryl N‐oxidation of amine‐containing molecules. Org Lett 20: 2011–2014. 29547294 10.1021/acs.orglett.8b00558 PMC6431535

[mbt213849-bib-0007] Ghaleb, A. , Aouidate, A. , Ayouchia, H.B.E. , Aarjane, M. , Anane, H. , and Stiriba, S.E. (2020) In silico molecular investigations of pyridine N‐Oxide compounds as potential inhibitors of SARS‐CoV‐2: 3D QSAR, molecular docking modeling, and ADMET screening. J Biomol Struct Dyn 17: 1–11. 10.1080/07391102.2020.1808530 32799761

[mbt213849-bib-0008] Gomila, M. , Peña, A. , Mulet, M. , Lalucat, J. , and García‐Valdés, E. (2015) Phylogenomics and systematics in *Pseudomonas* . Front Microbiol 18: 214. 10.3389/fmicb.2015.00214 PMC4447124 26074881

[mbt213849-bib-0009] Guo, S. , Liu, R. , Wang, W. , Hu, H. , Li, Z. , and Zhang, X. (2020) Designing an artificial pathway for the biosynthesis of a novel phenazine N‐oxide in *Pseudomonas chlororaphis* HT66. ACS Synth Biol. 17: 883–892. 10.1021/acssynbio.9b00515 32197042

[mbt213849-bib-0010] Hibi, M. , Fukuda, D. , Kenchu, C. , Nojiri, M. , Hara, R. , Takeuchi, M. , *et al*. (2021) A three‐component monooxygenase from *Rhodococcus wratislaviensis* may expand industrial applications of bacterial enzymes. Commun Biol. 4: 16. 33398074 10.1038/s42003-020-01555-3 PMC7782822

[mbt213849-bib-0011] Jovanovic, M.V. (1984) Syntheses of some pyrimidine N‐oxides. Can J Chem 62: 1176.

[mbt213849-bib-0012] Kutanovas, S. , Rutkienė, R. , Tauraitė, D. , and Meškys, R. (2013) Bioconversion of methylpyrazines and pyridines using novel pyrazines‐degrading microorganisms. Chemija 24: 67–73.

[mbt213849-bib-0013] Leahy, J.G. , Batchelor, P.J. , and Morcomb, S.M. (2003) Evolution of the soluble diiron monooxygenases. FEMS Microbiol Rev 27: 449–479. 14550940 10.1016/S0168-6445(03)00023-8

[mbt213849-bib-0014] Ma, Z. , Li, J. , Lin, K. , Ramachandran, M. , Li, M. , and Li, Y. (2020) Heterocyclic N‐oxides as small‐molecule fluorogenic scaffolds: rational design and applications of their "On‐Off" fluorescence. Anal Chem 92: 12282–12289. 32790290 10.1021/acs.analchem.0c01918

[mbt213849-bib-0015] Martínez‐García, E. , and de Lorenzo, V. (2019) *Pseudomonas putida* in the quest of programmable chemistry. Curr Opin Biotechnol 59: 111–121. 31048223 10.1016/j.copbio.2019.03.012

[mbt213849-bib-0016] Mfuh, A.M. , and Larionov, O.V. (2015) Heterocyclic N‐oxides – an emerging class of therapeutic agents. Curr Med Chem 22: 2819–2857. 26087764 10.2174/0929867322666150619104007 PMC4711945

[mbt213849-bib-0017] Mitsukura, K. , Hayashi, M. , Yoshida, T. , and Nagasawa, T. (2013) Oxidation of aromatic N‐heterocyclic compounds to N‐oxides by *Verticillium* sp. GF39 cells. J Biosci Bioeng 115: 651–653. 23290448 10.1016/j.jbiosc.2012.12.002

[mbt213849-bib-0018] Morgan, G.L. , and Li, B. (2020) *In vitro* reconstitution reveals a central role for the N‐oxygenase PvfB in (dihydro)pyrazine‐N‐oxide and valdiazen biosynthesis. Angew Chem Int Ed Engl 23: 21387–21391. 10.1002/anie.202005554 PMC7721988 32662921

[mbt213849-bib-0019] Narancic, T. , Salvador, M. , Hughes, G.M. , Beagan, N. , Abdulmutalib, U. , Kenny, S.T. , *et al*. (2021) Genome analysis of the metabolically versatile *Pseudomonas umsongensis* GO16: the genetic basis for PET monomer upcycling into polyhydroxyalkanoates. Microb Biotechnol. 1–18. 10.1111/1751-7915.13712 PMC8601165 33404203

[mbt213849-bib-0020] Nesi, R. , Giomi, D. , Papaleo, S. , and Turchi, S. (1992) Polyfunctionalized 3‐nitropyridine derivatives by [4 + 2] cycloadditions of 4‐nitro‐3‐phenylisoxazole‐5‐carboxylates with enamines: applications and limits. J Org Chem 57: 3713–3716.

[mbt213849-bib-0021] Nichol, T. , Murrell, J.C. , and Smith, T.J. (2015) Controlling the activities of the diiron centre in bacterial monooxygenases: lessons from mutagenesis and biodiversity. Eur J Inorg Chem 2015: 3419–3431.

[mbt213849-bib-0022] Nikel, P.I. , Chavarria, M. , Fuhrer, T. , Sauer, U. , and de Lorenzo, V. (2015) *Pseudomonas putida* KT2440 strain metabolizes glucose through a cycle formed by enzymes of the Entner‐Doudoroff, Embden‐Meyerhof‐Parnas, and pentose phosphate pathways. J Biol Chem 290: 25920–25932. 26350459 10.1074/jbc.M115.687749 PMC4646247

[mbt213849-bib-0023] Petkevičius, V. , Vaitekūnas, J. , Stankevičiūtė, J. , Gasparavičiūtė, R. , and Meškys, R. (2018) Catabolism of 2‐hydroxypyridine by *Burkholderia* sp. strain MAK1: a 2‐hydroxypyridine 5‐monooxygenase encoded by hpdABCDE catalyzes the first step of biodegradation. Appl Environ Microbiol 84: e00387–e418. 29602788 10.1128/AEM.00387-18 PMC5960968

[mbt213849-bib-0024] Petkevičius, V. , Vaitekūnas, J. , Tauraitė, D. , Stankevičiūtė, J. , Šarlauskas, J. , Čėnas, N. , and Meškys, R. (2019) A biocatalytic synthesis of heteroaromatic N‐oxides by whole cells of *Escherichia coli* expressing the multicomponent, soluble di‐iron monooxygenase (SDIMO) PmlABCDEF. Adv Synth Catal 361: 2456–2465.

[mbt213849-bib-0025] Poblete‐Castro, I. , Becker, J. , Dohnt, K. , dos Santos, V.M. , and Wittmann, C. (2012) Industrial biotechnology of *Pseudomonas putida* and related species. Appl Microbiol Biotechnol 93: 2279–2290. 22350258 10.1007/s00253-012-3928-0

[mbt213849-bib-0026] Sato, N. (1985) Studies on pyrazines. 12. N‐oxidation of aminopyrazines with m‐chloroperbenzoic acid. J Heterocycl Chem 22: 1145–1146.

[mbt213849-bib-0027] Srinivasarao, S. , Nandikolla, A. , Suresh, A. , Ewa, A.‐K. , Głogowska, A. , Ghosh, B. , *et al*. (2020) Discovery of 1,2,3‐triazole based quinoxaline‐1,4‐di‐N‐oxide derivatives as potential anti‐tubercular agents. Bioorg Chem 100: 103955. 32464405 10.1016/j.bioorg.2020.103955

[mbt213849-bib-0028] Staijen, I.E. , van Beilen, J.B. , and Witholt, B. (2000) Expression, stability and performance of the three‐component alkane mono‐oxygenase of *Pseudomonas oleovorans* in *Escherichia coli* . Eur J Biochem 267: 1957–1965. 10727934 10.1046/j.1432-1327.2000.01196.x

[mbt213849-bib-0029] Stankevičiūtė, J. , Vaitekūnas, J. , Petkevičius, V. , Gasparavičiūtė, R. , Tauraitė, D. , and Meškys, R. (2016) Oxyfunctionalization of pyridine derivatives using whole cells of *Burkholderia* sp. MAK1. Sci Rep. 6: 39129. 27982075 10.1038/srep39129 PMC5159870

[mbt213849-bib-0030] Sullivan, J.P. , Dickinson, D. , and Chase, H.A. (1998) Methanotrophs, *Methylosinus trichosporium* OB3b, sMMO, and their application to bioremediation. Crit Rev Microbiol 24: 335–373. 9887367 10.1080/10408419891294217

[mbt213849-bib-0031] Sun, J.‐Q. , Xu, L. , Tang, Y.‐Q. , Chen, F.‐M. , Zhao, J.‐J. , and Wu, X.‐L. (2014) Bacterial pyridine hydroxylation is ubiquitous in environment. Appl Microbiol Biotechnol 98: 455–464. 23519734 10.1007/s00253-013-4818-9

[mbt213849-bib-0032] Torres Pazmiño, D.E. , Winkler, M. , Glieder, A. , and Fraaije, M.W. (2010) Monooxygenases as biocatalysts: classification, mechanistic aspects and biotechnological applications. J Biotechnol 146: 9–24. 20132846 10.1016/j.jbiotec.2010.01.021

[mbt213849-bib-0033] Ullrich, R. , Dolge, C. , Kluge, M. , and Hofrichter, M. (2008) Pyridine as novel substrate for regioselective oxygenation with aromatic peroxygenase from *Agrocybe aegerita* . FEBS Lett 582: 4100–4106. 19022254 10.1016/j.febslet.2008.11.006

[mbt213849-bib-0034] Verhoef, S. , Wierckx, N. , Westerhof, R.G.M. , de Winde, J.H. , and Ruijssenaars, H.J. (2009) Bioproduction of *p*‐hydroxystyrene from glucose by the solvent‐tolerant bacterium *Pseudomonas putida* S12 in a two‐phase water‐decanol fermentation. Appl Environ Microbiol 75: 931–936. 19060171 10.1128/AEM.02186-08 PMC2643573

[mbt213849-bib-0035] Vörös, A. , Timári, G. , Baán, Z. , Mizsey, P. , and Finta, Z. (2014) Preparation of pyridine N‐oxide derivatives in microreactor. Period Polytech Chem. 58: 195–205.

[mbt213849-bib-0036] Wang, D. , Zhao, J. , Wang, Y. , Hu, J. , Li, L. , Miao, L. , *et al*. (2016) General and efficient synthesis of 2‐pyridones, 2‐quinolinones, and 1‐isoquinolinones from azine N‐oxides. Asian J Org Chem. 5: 1442–1446.

[mbt213849-bib-0037] Wrzeszcz, Z. , and Siedlecka, R. (2020) Heteroaromatic N‐oxides in asymmetric catalysis: a review. Molecules 25: 330. 10.3390/molecules25020330 PMC7024222 31947566

