# Supplementary figures and images for: An efficient and regioselective biocatalytic synthesis of aromatic N‐oxides by using a soluble di‐iron monooxygenase PmlABCDEF produced in the Pseudomonas species
Source: Microb Biotechnol. 2021 Jun 11;14(4):1771–83. doi: 10.1111/1751-7915.13849 (PMC8313251; doi:10.1111/1751-7915.13849)

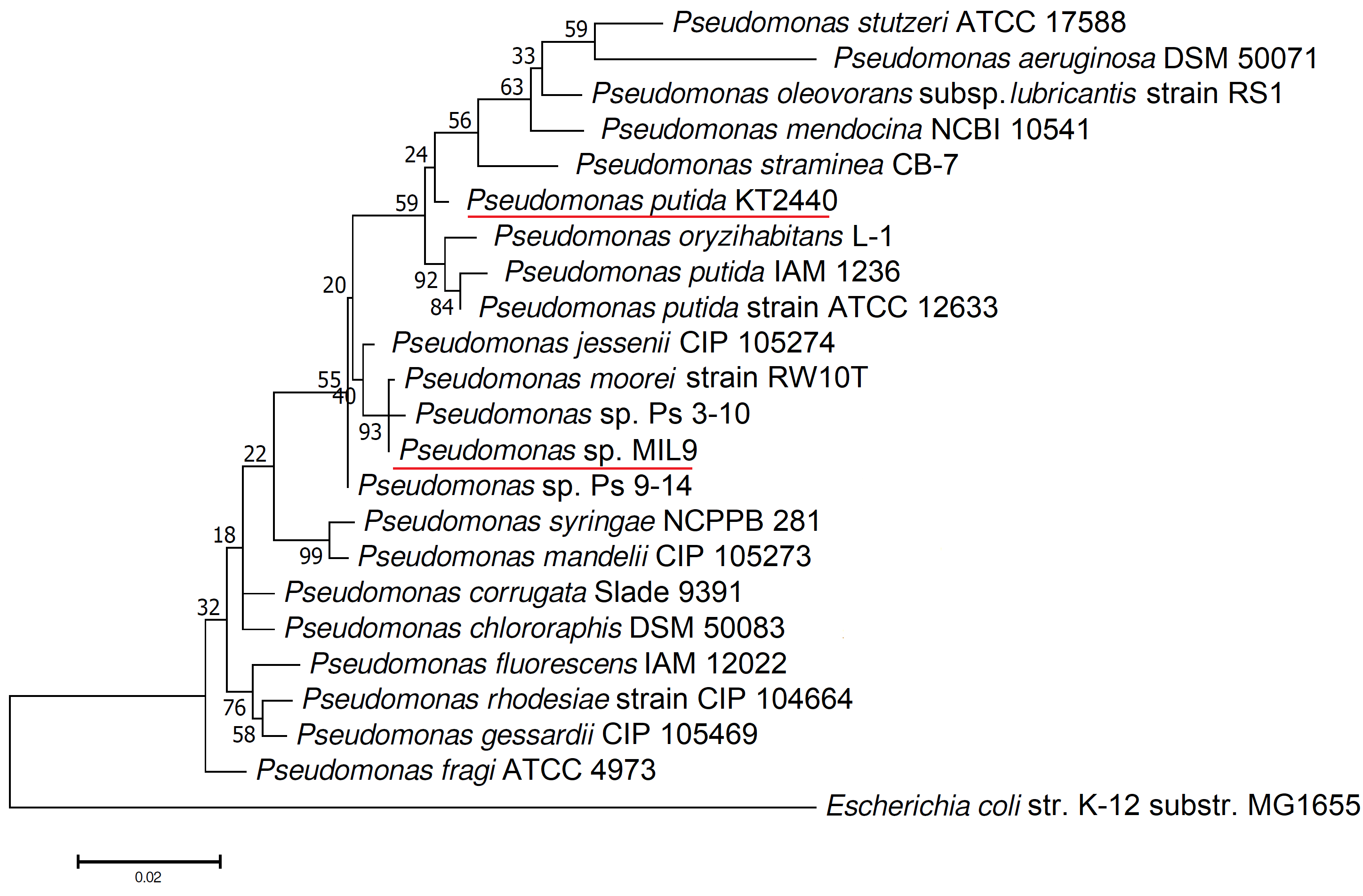

Supplement: Supplementary file 1 — Fig. S1. Maximum‐likelihood phylogenetic tree based on the partial 16S rRNA gene sequences of members of the Pseudomonas genus. The percentage of 500 trees in which the associated taxa clustered together after a bootstrap analysis is shown next to the branches. The 16S rRNA sequence of E. coli K12 was used as an outgroup. [file MBT2-14-1771-s002.tif]

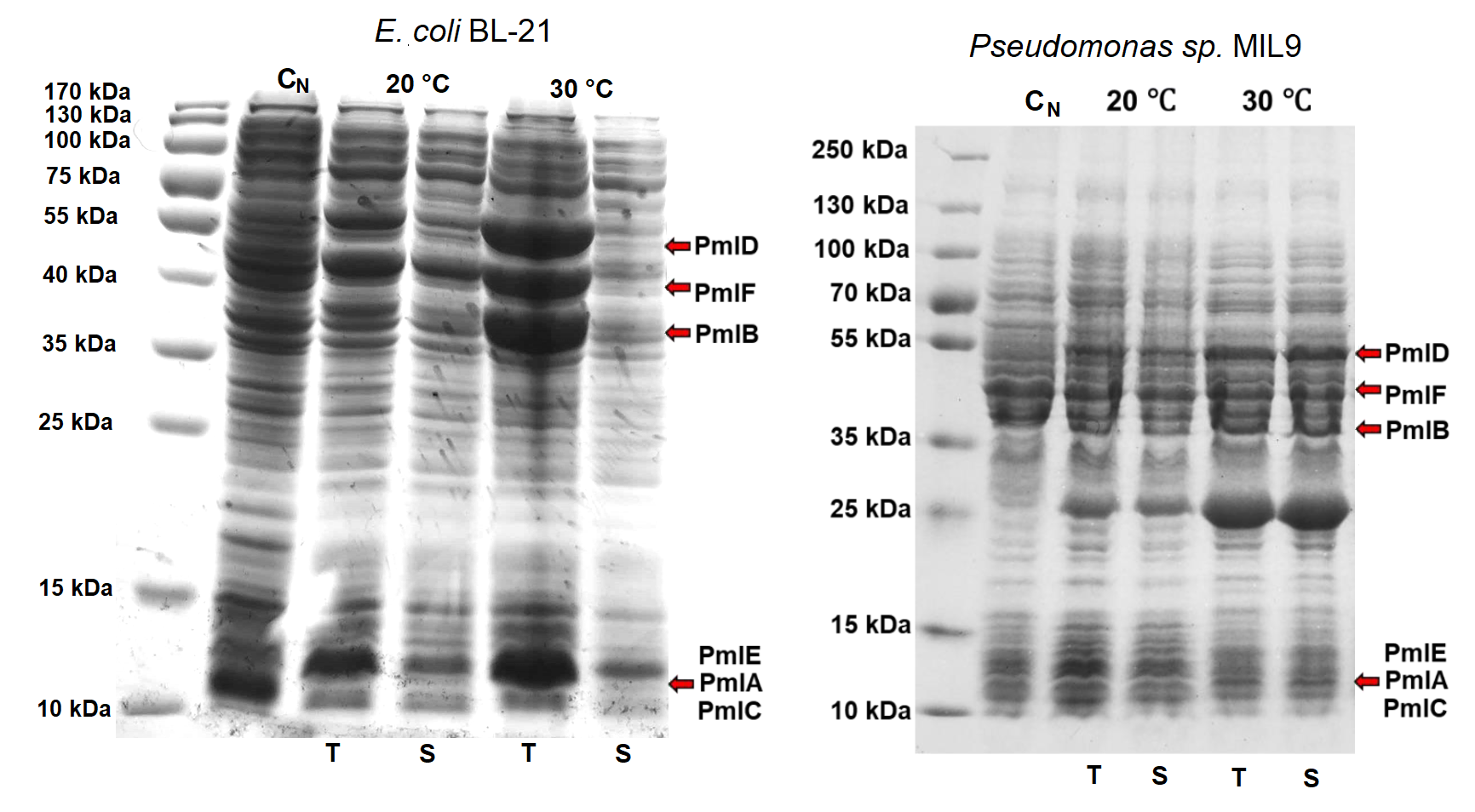

Supplement: Supplementary file 2 — Fig. S2. PmlABCDEF biosynthesis analysis in different hosts. Calculated molecular weight of individual PmlABCDEF subunits: PmlA – 11 kDa, PmlB – 38 kDa, PmlC – 10 kDa, PmlD – 59 kDa, PmlE – 13 kDa, PmlF – 39 kDa. Negative controls (CN) is a cell‐free extract of E. coli BL‐21 and Pseudomonas sp. MIL9 respectively. (T) indicates a total fraction of cell‐free extract, (S) – soluble fraction. [file MBT2-14-1771-s001.tif]
